# Prolonged mechanical ventilation after lung transplantation: risks factors and consequences on recipient outcome

**DOI:** 10.3389/fmed.2023.1160621

**Published:** 2023-05-09

**Authors:** Enora Atchade, Aimane Boughaba, Alexy Tran Dinh, Sylvain Jean-Baptiste, Sébastien Tanaka, Léa Copelovici, Brice Lortat-Jacob, Arnaud Roussel, Yves Castier, Jonathan Messika, Hervé Mal, Christian de Tymowski, Philippe Montravers

**Affiliations:** ^1^APHP, CHU Bichat-Claude Bernard, DMU PARABOL, Paris, France; ^2^INSERM U1148, LVTS, CHU Bichat-Claude Bernard, Paris, France; ^3^Université de Paris, UFR Diderot, Paris, France; ^4^Université De La Réunion, INSERM UMR 1188, Diabète Athérothrombose Réunion Océan Indien (DéTROI), Saint-Denis de la Réunion, France; ^5^APHP, CHU Bichat-Claude Bernard, Service de Chirurgie Thoracique et Vasculaire, 46 rue Henri Huchard, Paris, France; ^6^INSERM UMR 1152, Physiopathologie et Epidémiologie des Maladies Respiratoires, Paris, France; ^7^APHP, CHU Bichat-Claude Bernard, Service de Pneumologie B et Transplantation Pulmonaire, Paris, France; ^8^INSERM UMR 1149, Immunorecepteur et Immunopathologie Rénale, CHU Bichat-Claude Bernard, Paris, France

**Keywords:** lung transplantation, intensive care unit, prolonged mechanical ventilation, outcome, one-year mortality

## Abstract

**Background:**

Risk factors and the incidence of prolonged mechanical ventilation (PMV) after lung transplantation (LT) have been poorly described. The study assessed predictive factors of PMV after LT.

**Methods:**

This observational, retrospective, monocentric study included all patients who received LT in Bichat Claude Bernard Hospital between January 2016 and December 2020. PMV was defined as a duration of MV > 14 days. Independent risk factors for PMV were studied using multivariate analysis. One-year survival depending on PMV was studied using Kaplan Meier and log-rank tests. A *p* value <0.05 was defined as significant.

**Results:**

224 LT recipients were analysed. 64 (28%) of them received PMV for a median duration of 34 [26–52] days versus 2 [1–3] days without PMV. Independent risk factors for PMV were higher body mass index (BMI) (*p* = 0.031), diabetes mellitus of the recipient (*p* = 0.039), ECMO support during surgery (*p* = 0.029) and intraoperative transfusion >5 red blood cell units (*p* < 0.001). Increased mortality rates were observed at one-year in recipients who received PMV (44% versus 15%, *p* < 0.001).

**Conclusion:**

PMV was associated with increased morbidity and mortality one-year after LT. Preoperative risk factors (BMI and diabetes mellitus) must be considered when selecting and conditioning the recipients.

## Background

Ten percent of patients who require mechanical ventilation (MV) receive prolonged mechanical ventilation (PMV) (1). The mortality rates of these patients are high and were approximately 29% at hospital discharge and 59 to 62% at one-year in a recent meta-analysis (2). Sepsis, metabolic disorders (including malnutrition), cardiovascular failure, neuromyopathy, cognitive impairment, diaphragmatic dysfunction, bronchial or tracheal obstruction, and ineffective cough are risk factors for PMV (3).

Only one prior retrospective study assessed the causes and impact of PMV in lung transplantation (LT) (4). This study analysed a monocentric cohort of 690 LT recipients between 2005 and 2012, including 41 children. The incidence of PMV (defined as MV > 21 days) was 13.8%. Risk factors for PMV in this study were renal replacement therapy (RRT) (OR 11.13, *p* < 0.001), bronchial anastomotic dehiscence (OR 8.74, *p* = 0.001), autoimmune comorbidities (OR 5.52, *p* = 0.002) and postoperative neurological complications (OR 5.03, *p* = 0.001). Survival after hospital discharge was lower in PMV recipients (67% versus 95% without PMV), and one-year survival decreased (60.7% versus 90.0% without PMV, *p* < 0.001).

Recently, Hu et al. have reported risks factors of PMV in a retrospective monocentric cohort of 1,138 LT recipients (5). In this analysis, the threshold to define PMV was 60 days. Independent risk factors for PMV were bridging on MV, double lung transplantation, concomitant cardiac procedure, re-exploration for bleeding, and primary graft dysfunction higher grades.

The present study assessed independent risk factors for PMV after LT. We also described the incidence of PMV and the outcome of LT recipients (postoperative complications and one-year mortality) depending on PMV.

## Methods

### Study population

This retrospective, observational study assessed all patients who underwent LT in Bichat-Claude Bernard Hospital in Paris between January 2016 and December 2020. Patients who died before day 15 were excluded from the analysis. The Paris North Hospital Institutional Review Board (Paris Diderot University, Assistance Publique Hôpitaux de Paris No. 0007477) reviewed and approved the study.

### Perioperative management

Perioperative management of LT is standardised in our centre, according to our local protocol (6, 7) and current practises (8). After lung removal, graft preservation was performed by administration of preservation fluid (Perfadex ®) –4 l anterograde administration followed by 2 l retrograde administration. The grafts were then stored in a transport bag with 1 l of preservation fluid. Before surgery, an epidural anaesthesia catheter was inserted in the absence of contraindications. General anaesthesia was performed using propofol, remifentanil, and neuromuscular blocking agents. In the absence of randomised controlled trials assessing ventilation strategies in LT recipients and based on the available indirect evidence (9, 10), lung-protective MV (low tidal volumes (≤ 6 ml/kg of predicted body weight of the recipient) and positive end-expiratory pressure) was administered during and after the surgical procedure. Extracorporeal membrane oxygenation (ECMO) support was used in cases of severe pulmonary hypertension, pre-existing or perioperative right-sided cardiac dysfunction, or when the patient did not tolerate single-lung ventilation. The bronchial anastomosis technique was performed as follows: on the left side, a limited dissection of the recipient bronchus and a section close to the mediastinum was performed. On the right side, a limited dissection of the recipient bronchus and a section at one ring or less of the birth of the upper lobar bronchus was performed. Anastomosis was performed without intussusception and buried in the peribronchial lymph node tissue on the right side and under the posterior pericardium on the left side. Intraoperative endoscopic control of bronchial anastomosis was systematically performed. During the surgical procedure, invasive arterial pressure, Swan Ganz catheter, and transoesophageal echocardiography were used for haemodynamic monitoring and optimisation of fluid administration. A restrictive transfusion threshold (haemoglobin concentration < 7 g/dL) was used for red blood cell (RBC) transfusion (7). An autotransfusion system (Cell-saver®, Fresenius, Bad Homburg vor der Höhe, Germany) was used, and tranexamic acid was administered in the absence of contraindications. Antibiotic prophylaxis consisted of systematic administration of first-generation cephalosporin for at least 48 h, which was secondarily adapted in cases of prior colonisation of the recipient or pneumonia of the donor. Before extubation, a weaning trial was performed with spontaneous breathing in the T-piece for 30 min. Weaning trial success was defined as an SpO2 > 92% and respiratory frequency between 10 and 30 cycles per minute at the end of the trial without agitation, hypertension or tachycardia (11). After extubation, non-invasive ventilation was systematically used. As early tracheostomy is not associated to a lower mortality in the ICU patients receiving mechanical ventilation compared to delayed or no tracheostomy (12), tracheostomy was performed in LT recipients when it seemed able to facilitate ventilation weaning, and when the clinical situation allowed the procedure. Iterative bronchoscopies were performed every 48 h in the early postoperative period to search for bronchial complications or pneumonia. The definition of pneumonia is standardised (13). The duration of antibiotic therapy in cases of pneumonia was 7 days. Immunosuppressive therapy was based on a combination of prednisolone, tacrolimus, and mycophenolate mofetil.

### Data collection

Data linked to the recipients (e.g., demographic data, comorbidities, and underlying disease), donors (e.g., age, PaO2/FiO2, tobacco use, transfusion, cause of death, antibiotic treatment), and surgical procedures (e.g., type of LT, need for a high emergency procedure, ECMO or catecholamine support, vascular filling, transfusion, and epidural anaesthesia) were prospectively collected. After the surgical procedure, the following elements were recorded: severity scores on admission to the ICU (SOFA and SAPS II score), respiratory status during the ICU stay (duration of MV, prone positioning, neuromuscular agent administration, number of pneumonia episodes, primary graft dysfunction (PGD) and grade at H24, H48, H72, and tracheostomy for ventilation weaning), and postoperative complications (multiorgan failure syndrome, septic shock, duration of catecholamine and ECMO support, acute kidney injury (AKI), renal replacement therapy (RRT), acute cellular rejection, antibody mediated rejection, and surgical reintervention). The outcome of the recipients (death at day 90 and one-year and duration of hospitalisation in ICU) was assessed.

### Definitions

PMV was defined as the need for ventilator support on the tracheal tube or tracheal cannula for 15 days or more. The main aetiologies of PMV were stratified as parenchymal, pleural, bronchial, diaphragmatic, cardiogenic and neurological causes, and several aetiologies were recorded for the same patient (3, 14, 15). Pneumonia was defined as positive culture of bronchoalveolar lavage (BAL) samples that yielded ≥10^4 colony-forming units (CFU)/mL combined with new or persistent lung infiltrate on chest X-ray, and two or more of the following criteria: temperature ≥ 38.4°C or < 36°C, WBC count >11,000/mm3 or < 4,000/mm3, at least 30% decrease in PaO2/FiO2 ratio, and purulent secretions (13). Extubation failure was defined as the need for reintubation during the first 72 h following extubation (11). AKI was defined according to the Kidney Disease: Improving Global Outcomes (KDIGO) criteria (16). Primary graft dysfunction (PGD) was defined according to the ISHLT criteria (17), and septic shock was defined according to the Sepsis-3 definition (18).

### Statistical analysis

Qualitative data are expressed as absolute numbers and percentages, and quantitative data are expressed as medians and interquartile ranges [IQRs]. Variables were first analysed using univariate analyses (Fisher’s exact tests, χ2, and Mann–Whitney tests, as appropriate). A multivariate analysis was performed to assess independent risk factors for PMV and one-year mortality. Variables with a *p* value <0.2 in univariate analysis were entered in a backward stepwise logistic regression model to identify independent risk factors. Ninety-day and one-year survival were assessed using Kaplan–Meier curves and compared using log rank tests. The third tertile of PMV recipients was compared to the other recipients to assess specific risk factors for extended MV and study the perioperative complications and outcomes of these patients. A *p* value <0.05 was defined as statistically significant. RCore Team (2013) was used for statistical analyses (R Foundation for Statistical Computing, Vienna, Austria, http://www.R-project.org/).

## Results

### Characteristics of the study population

**Figure 1:**
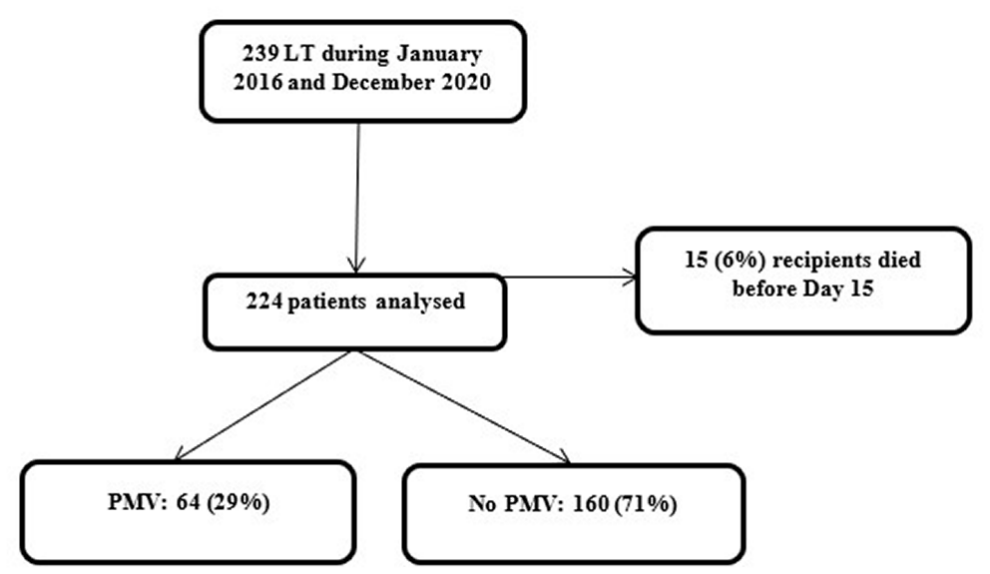
Flow chart of the study.

A total of 239 patients underwent LT in our institution between January 2016 and December 2020. Fifteen (6%) of them died before day 15 and were excluded from the analysis. A total of 224 patients were analysed. The flow chart of the study is presented in Figure 1. Recipients were primarily men (146 (65%) patients), with a median age of 56 [50–62] years (Table 1). Predominant pulmonary underlying diseases were pulmonary fibrosis (PF) (80 (36%) patients) and chronic obstructive pulmonary disease (COPD) (60 (27%) patients). The median duration of MV for the cohort was 3 [1–19] days, and the median duration of hospitalisation in the ICU was 17 [11–32] days. Mortality rates at day 90 and one-year were 15 and 28%, respectively. Overall, 160 (71%) patients required ECMO support during surgery (veno-arterial support in 156 patients (98% of the patients who required peroperative ECMO support) and veno-venous support in 4 patients (2% of the patients who required ECMO support during surgery)). Cannulation sites were femoro-femoral in a huge majority of the cases (138 patients (86% of the patients who required peroperative ECMO)), axillo-femoral in 7 patients (4%), jugulo-femoral in 3 patients (2%), aorto-femoral in 2 patients (1%), jugulo-femoro-femoral in 2 patients (1%).

**Table 1:** Risks factors for PMV identified in univariate and multivariate analyses.

				Univariate analysis		Multivariate analysis
	All patients *n* = 224 (100%)	PMV *n* = 64 (29%)	No PMV *n* = 160 (71%)	*p*	OR	95%CI	*p* value
*Preoperative variables*							
Age (recipient), years, median [IQR]	56 [50–62]	56 [45–59]	56 [51–63]	0.039	–	–	–
Male gender, *n* (%)	146 (65)	38 (59)	108 (68)	0.25	–	–	–
Diagnosis leading to LT, *n* (%)							
Pulmonary fibrosis	80 (36)	23 (36)	57 (36)	0.97	–	–	–
COPD	60 (27)	12 (19)	48 (30)	0.086	–	–	–
BMI, median [IQR]	24 [20–27]	25 [22–28]	23 [20–26]	0.004	1.09	1.01–1.17	0.031
BMI < 18.5 Kg/m^2^	33 (15)	7 (11)	26 (16)	0.015			
BMI [18.5–24.9] Kg/m^2^	93 (42)	18 (29)	75 (47)				
BMI [25–29.9] Kg/m^2^	67 (30)	25 (40)	42 (26)				
BMI ≥ 30 kg/m2	30 (13)	13 (20)	17 (11)		–	–	–
Comorbidities							
High blood pressure, *n* (%)	56 (25)	17(27)	39 (24)	0.73	–	–	–
Pulmonary hypertension, *n* (%)	115 (52)	27 (44)	88 (55)	0.14	–	–	–
Diabetes mellitus, *n* (%)	23 (10)	11 (17)	12 (8)	0.031	3.02	1.05–8.80	0.039
HbA1c of diabetic patients, %, median [IQR]	6.5 [6.1–7.2]	6.5 [6.3–7.3]	6.6 [6.2–7.0]	1			
Dyslipidaemia, *n* (%)	48 (21)	9 (14)	39 (24)	0.089	–	–	–
Serum albumin, median [IQR]	40 [36–44]	39 [37–42]	40 [36–44]	0.42	–	–	–
Serum creatinine, median [IQR]	69 [54–82]	67 [56–87]	70 [54–80]	0.82	–	–	–
Pulmonary hypertension, *n* (%)	115 (52)	27 (44)	88 (55)	0.14	–	–	–
Dilatation of the right ventricle, *n* (%)	59 (26)	15 (23)	44 (28)	0.53	–	–	–
Moderate dilatation, *n* (%)	48 (22)	14 (23)	34 (21)	0.44			
Severe dilatation, *n* (%)	11 (5)	1 (2)	10 (6)		–	–	–
Re-transplantation, *n* (%)	5 (2)	3 (5)	2 (1)	0.14		–	–
High emergency LT, *n* (%)	41 (18)	18 (28)	23 (14)	0.016		–	–
ECMO support before surgery, *n* (%)	17 (8)	9 (14)	8 (5)	0.027		–	–
MV before surgery, *n* (%)	7 (3)	5 (8)	2 (1)	0.021		–	–
High flow oxygen therapy before surgery, *n* (%)	35 (16)	17 (27)	18 (11)	0.004		–	–
Donor variables							
Male gender, *n* (%)	124 (55)	33 (52)	91 (57)	0.74 *0.025*	–	–	–
PaO2/FiO2 ratio, median [IQR]	390 [330–454]	384 [329–429]	400 [333–471]	0.17	1.00	0.99–1.00	0.033
Age (donor), median [IQR]	51 [38, 60]	55 [44, 60]	48 [34, 59]	0.028	1.03	1.00–1.05	0.024
Tobacco use (donor), n (%)	86 (39)	30 (49)	56 (35)	0.062	–	–	–
Transfusion of the donor, n (%)	62 (28)	19 (31)	43 (27)	0.61	–	–	–
Duration of MV, median [IQR]	2 [1–3]	2 [1–4]	2 [1–3]	0.43	–	–	–
Cause of death							
Trauma, *n* (%)	65 (29)	19 (30)	46 (29)	0.87			
Cardiac arrest, *n* (%)	50 (22)	15 (23)	35 (22)	0.72			
Stroke or intracranial bleeding process, *n* (%)	131 (58)	44 (69)	87 (54)	0.052			
Antibiotic treatment, *n* (%)	134 (60)	34 (53)	100 (63)	0.23			
Amoxicillin / clavulanate, *n* (%)	107 (48)	29 (45)	78 (49)	0.66			
Intraoperative variables							
Bilateral LT, *n* (%)	157(70)	469 (77)	108 (68)	0.18	–	–	–
Cold ischemia time, min, median [IQR]							
Unilateral LT or first lung in bilateral LT	278 [235–330]	275 [235–335]	280 [236–330]	0.64			
Second lung in bilateral LT	360 [300–424]	360 [304–435]	360 [300–420]	0.30			
Duration of anaesthesia, min, median [IQR]	420 [360, 480]	420 [360, 510]	420 [345, 480]	0.19			
Epidural anaesthesia, *n* (%)	144 (65)	37 (59)	107 (68)	0.21	–	–	–
Haemodynamic support by ECMO, *n* (%)	160 (71)	57 (89)	103 (64)	<0.001	2.83	1.16–7.73	0.029
Veno-arterial support, *n* (%)	156 (70)	56 (87)	100 (62)	0.0089			
Veno-venous support, *n* (%)	4 (1)	1 (2)	3 (2)	1			
Norepinephrine or epinephrine >0.5 μg/kg/min, *n* (%)	74 (34)	32 (51)	42 (27)	< 0.001	–	–	–
Vascular filling >2,500 mL	192 (86)	57 (89)	135 (85)	0.48			
Red blood cell transfusion, *n* (%)	145(65)	51 (80)	94 (59)	0.004	–	–	–
Transfusion >5 PRC, *n* (%)	35 (16)	22 (34)	13 (8)	<0.001	6.74	2.81–17.20	<0.001
Fresh frozen plasma transfusion, *n* (%)	134 (60)	47 (73)	87 (54)	0.009	–	–	–
Platelet transfusion, *n* (%)	45 (20)	20 (31)	25 (16)	0.008	–	–	–
At admission to ICU							
SOFA	7 [5–8]	8 [6–10]	6 [5–8]	0.001			
SAPS II	39 [28–48]	43 [32–52]	38 [28–46]	0.007			
Lactate >3 mmol/L	63 (28)	28 (44)	35 (22)	0.001			
Lactate >2 mmol/L	125(56)	42 (66)	83 (52)	0.061			

BMI, Body mass index; COPD, chronic obstructive pulmonary disease; ECMO, Extra-Corporeal Membrane Oxygenation; HBP, High blood pressure; ICU, intensive care unit; LT, lung transplantation; MV, mechanical ventilation; OR, odds ratio; PMV, prolonged mechanical ventilation; PRC, Packed Red Cell; SOFA, sequential organ failure assessment; SAPS, simplified acute physiology score.

### Incidence of PMV

**Figure 2:**
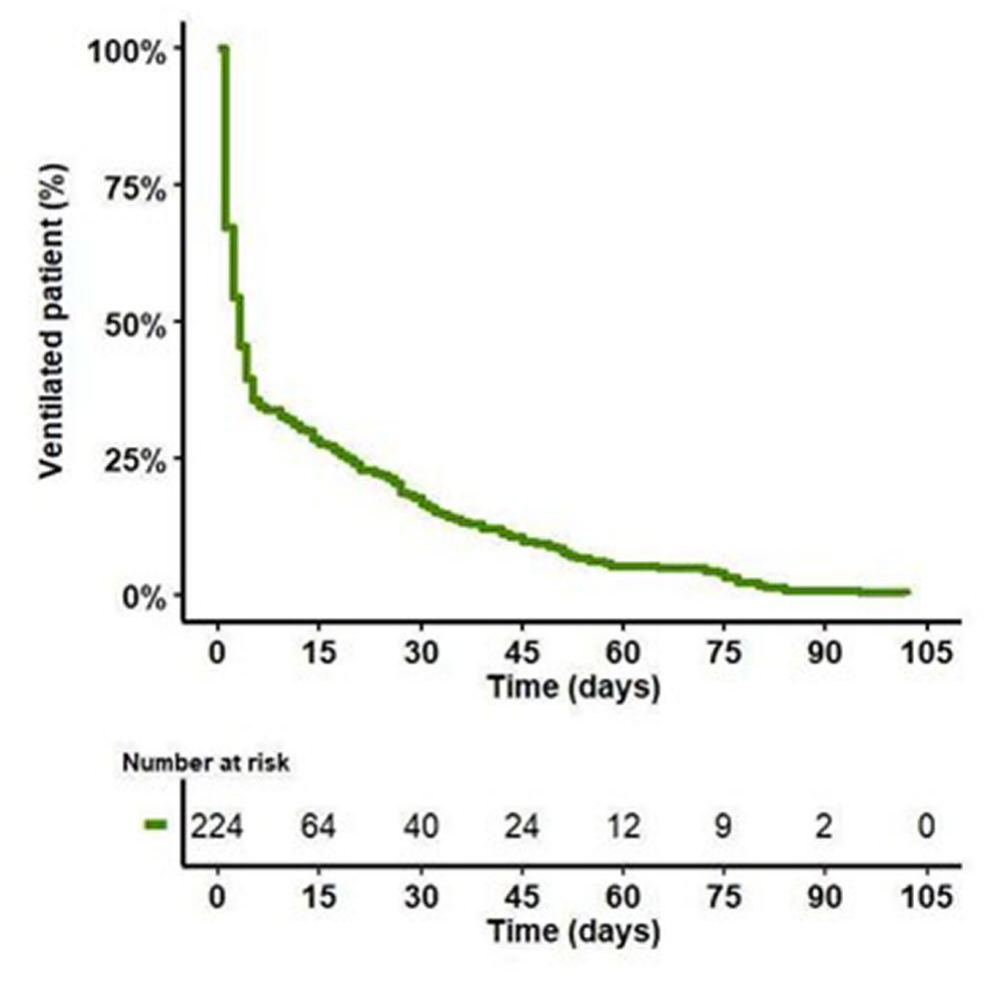
Evolution of the percentage of ventilated LT recipients after surgical procedure.

PMV occurred in 64 (29%) LT recipients. The median duration of MV was 34 [26–52] days in the PMV group versus 2 [1–3] days in the non-PMV group (*p* < 0.001). A total of 51 (23%) LT recipients underwent an MV duration >21 days. Fifty-eight (90%) of the PMV patients underwent tracheostomy to facilitate ventilation weaning after a median delay of 15 [13–20] days. The median duration of MV was 36 [26–53] in the recipients who underwent tracheostomy for ventilation weaning. The evolution of the percentage of ventilated LT recipients after surgical procedures is presented in Figure 2.

### Aetiologies of PMV and median duration of MV

The causes of PMV were multifactorial in all cases: neurological factors for 40 (63%) recipients, parenchymal factors in 64 (100%) recipients, bronchial factors for 37 (58%) recipients, pleural factors for 41 (64%) recipients, cardiogenic factors for 29 (45%) recipients, and diaphragmatic dysfunction for 17 (27%) recipients. The median duration of MV was not different when diaphragmatic dysfunction was considered a cause of PMV ((34 [28–47]) days versus 37 [25–57] days, *p* = 0.77). When a neurological disorder was a cause of PMV, the median duration of MV was significantly longer (39 [30–63] days versus 28 [20–43] days in the absence of neurological disorder, *p* = 0.01).

### Risk factors for PMV

Risk factors for PMV in univariate and multivariate analyses are presented in Table 1. Multivariate analysis revealed that higher body mass index (BMI) (OR 1.09, *p* = 0.031) and diabetes mellitus of the recipient (OR 3.02, *p* = 0.039), ECMO support during surgery (OR 2.83, *p* = 0.029), and intraoperative transfusion ≥5 RBC units (OR 6.74, *p* < 0.001) were independent risk factors for PMV.

### Postoperative complications and outcome depending on PMV of the recipients

Postoperative complications and outcome of the LT recipients depending on PMV in univariate analysis are presented in Table 2. All postoperative complications (duration of ECMO and catecholamine support, infectious complications, grade 3 PGD at H72, AKI and RRT requirement, and acute rejection) were significantly associated with PMV. These complications resulted in an increased duration of hospitalisation in the ICU (*p* < 0.001). The median duration of MV was 2 [1–5] days when no PGD occurred during the first 72 h, 1 [1–3] day in patients with grade 1 PGD, 3 [2–25] days in patients with grade 2 PGD, and 20 [4–35] days in patients with grade 3 PGD (*p* < 0.001). When comparing recipients with grade 3 PGD to recipients with grade 2 PGD, the odds ratio of PMV was 2.8 (*p* = 0.041).

**Table 2:** Postoperative complications and outcome of the LT recipients depending on PMV, univariate analysis.

	Entire cohort *n* = 224 (100%)	PMV *n* = 64 (29%)	No PMV *n* = 160 (71%)	*p*
*Postoperative complications*				
Multiorgan failure syndrome, *n* (%)	54 (26)	41 (64)	16 (10)	<0.001
Median duration of catecholamine support, days, median [IQR]	2 [1–4]	5 [2–12]	1 [1–2]	<0.001
ECMO support after surgery for PGD, *n* (%)	61 (27)	35 (55)	26 (16)	<0.001
Median duration of ECMO support, days, median [IQR]	0 [0–1]	1 [0–3]	0 [0–0]	<0.001
Septic shock, *n* (%)	56 (25)	45 (70)	11 (7)	<0.001
Number of pneumonia, median [IQR]	1 [1–2]	2 [1–3]	1 [1–1]	<0.001
Bacteriemia, *n* (%)	29 (13)	19 (31)	10 (6)	<0.001
Prone positioning, *n* (%)	34 (15)	28 (44)	6 (4)	<0.001
PGD during the first 72 h, *n* (%)				< 0.001
Grade 1	27 (12)	6 (10)	21 (4)	
Grade 2	22 (10)	7 (11)	15 (10)	
Grade 3	53 (25)	30 (48)	23 (15)	
Grade 3 PGD at H24, *n* (%)	51 (23)	25 (39)	26 (16)	<0.001
Grade 3 PGD at H48, *n* (%)	34 (15)	22 (34)	12 (8)	<0.001
Grade 3 PGD at H72, *n* (%)	29 (13)	20 (31)	9 (6)	<0.001
Use of neuromuscular blocking agent, *n* (%)	60 (27)	46(72)	14 (9)	<0.001
Atrial fibrillation, *n* (%)	79 (36)	34 (53)	45 (29)	<0.001
AKI, *n* (%)	97 (43)	46 (72)	51 (32)	<0.001
Renal replacement therapy, *n* (%)	17 (8)	13 (20)	4 (3)	<0.001
Thoracic surgical reintervention, *n* (%)	39 (17)	27(42)	12 (8)	<0.001
Abdominal surgery, *n* (%)	21 (9)	14 (22)	7 (4)	<0.001
Antibody mediated rejection, *n* (%)	67(30)	31 (48)	36 (22)	<0.001
Acute cellular rejection, *n* (%)	29 (13)	16 (25)	13 (8)	<0.001
*Outcome*				
Duration of MV, median [IQR]	3 [1–19]	34 [26–52]	2 [1–3]	<0.001
Duration of ICU stay, median [IQR]	17 [11–23]	52 [35–78]	13 [9–19]	<0.001
Death at day 90, *n* (%)	22 (10)	14 (22)	8 (5)	<0.001
Death at 1 year, *n* (%)	52 (23)	28 (44)	24 (15)	<0.001

AK, acute kidney injury; ECMO, extracorporeal membrane oxygenation; ICU, intensive care unit; PMV, prolonged mechanical ventilation; PGD, Primary graft dysfunction; RRT, renal replacement therapy.

### Risk factors for one-year mortality

**Figure 3:**
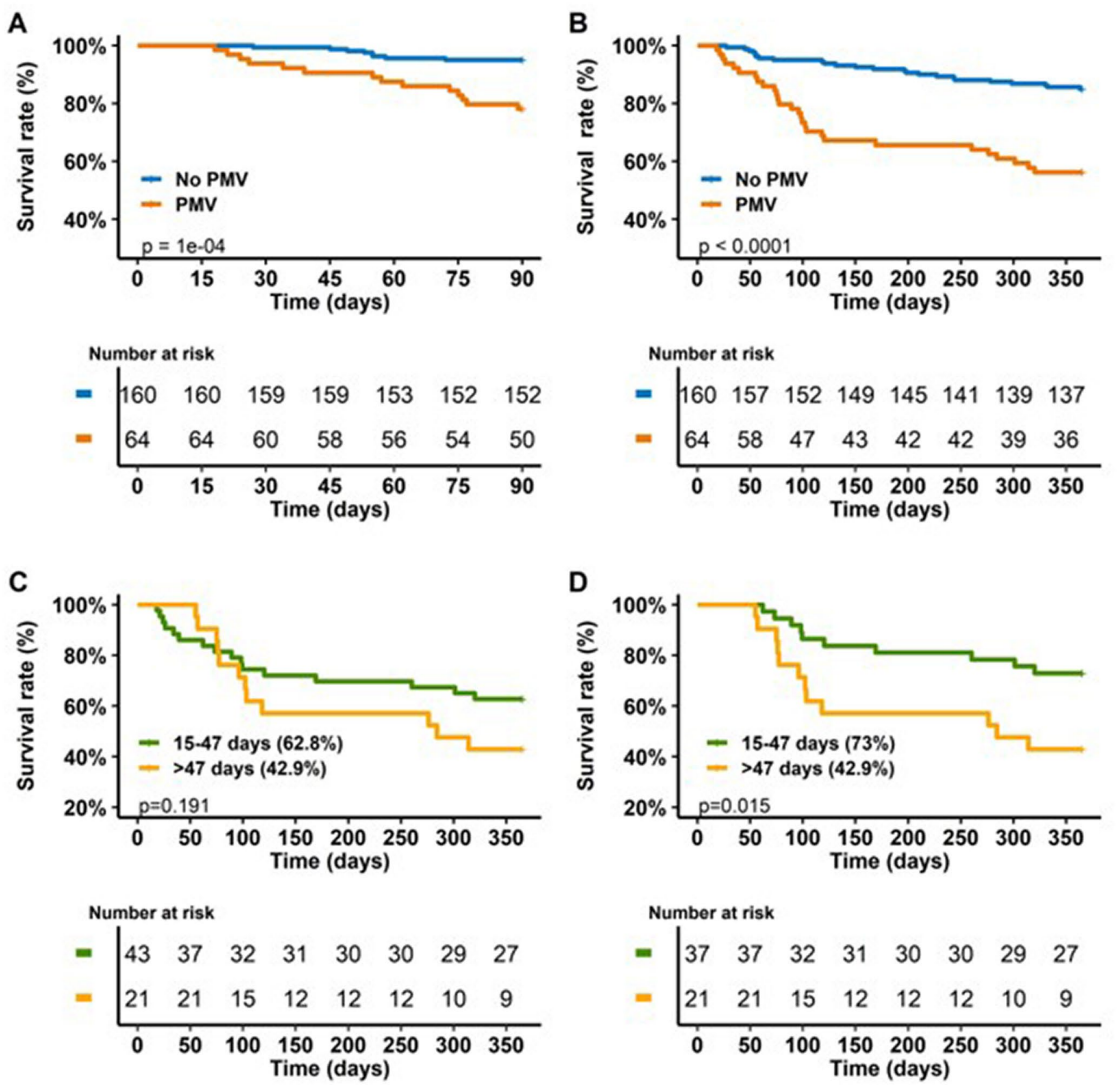
**(A,B)** 90 days and one-year survival of LT recipients depending on PMV. **(C)** One-year survival of PMV LT recipients depending on MV duration. **(D)** One-year survival of PMV LT recipients depending on MV duration after exclusion of patients who died before day 47.

Risk factors for one-year mortality in multivariate analysis are presented in Table 3. PMV was an independent risk factor for mortality at one-year (OR = 7.89, p < 0.001). The one-year survival rates of LT recipients depending on PMV and MV duration are presented in Figures 3A–D.

**Table 3:** Risk factors for death at 1 year, multivariate analysis.

	OR	95%CI	*p*
Male gender, recipient	5.83	2.25–16.97	0.001
Tobacco use, recipient	0.37	0.15–0.88	0.026
Peripheral artery occlusion disease	22.02	3.30–193.28	0.002
Age of the donor	1.04	1.01–1.07	0.013
Transfusion of the donor	2.66	1.08–6.75	0.034
Vascular filling >2,500 ml during surgery	13.57	2.28–268.64	0.019
Number of pneumonia	0.60	0.39–0.92	0.020
AKI	2.33	1.00–5.54	0.051
PMV	7.89	2.73–24.91	<0.001

AKI, acute kidney injury; PMV, prolonged mechanical ventilation.

### Focus on the third tertile of PMV patients: risk factors, perioperative complications and outcome of the recipients

The duration of MV in the third tertile of PMV recipients (21 (33%) PMV recipients) was >47 days. Risk factors for MV > 47 days are presented in Supplementary Table S1. Except for FFP and platelet transfusions during surgery, preoperative variables, intraoperative variables and severity scores at admission to the ICU were not significantly different between the two groups. Postoperative complications and outcomes of the third tertile of PMV recipients are presented in Supplementary Table S2.

## Discussion

Predictive factors of PMV after LT, postoperative complications and the outcomes of LT recipients depending on PMV of the recipients were studied in a retrospective monocentric cohort of 224 LT recipients. Multivariate analysis revealed that the risk factors for PMV linked to the recipient were higher BMI and diabetes mellitus. Predictive factors linked to the intraoperative period were ECMO support during surgery and transfusion of 5 RBC units or more during surgery. All postoperative complications were significantly more frequent in recipients with PMV. The 90 day and one-year mortality rates were higher in the PMV group. PMV was an independent risk factor for one-year mortality.

In the absence of a consensual definition of PMV in the literature, a threshold of 15 days or more was used in our study to define PMV. This threshold seemed to be clinically pertinent, as median duration of mechanical ventilation in the whole cohort was only 3 [1–19] days. Only 13 patients who required MV for 15 days or more were weaned from ventilator before day 21. Moreover, the results of our study confirmed the relevance of a 15-days threshold to define PMV, since patients who required MV for 15 days or more were at higher risk for one-year mortality and postoperative complications during hospitalisation in ICU. This effect on mortality might have been observed in patients who required MV for more than 21 days, but this trend was already observed after 15 days of MV. Finally, a previous study showed that more than 37 different definitions of PMV have been used in published studies on this topic, and the duration of MV was between 3 days and 3 months. A threshold of 21 days or more was used in only 12.6% of the prior studies, and the National Association for Medical Direction of Respiratory Care definition of MV > 21 days for at least 6 h a day was only applied in 6.7% of the studies (19).

A 29% incidence of PMV was observed in our cohort, which was higher than the 13.8% incidence described by Hadem et al. (4). This discrepancy may be explained by the definition of PMV used in our study (≥15 days versus 21 days in the Hadem study), the higher median age of our recipients (56 [50–62] versus 49 [35–57] years) and the smaller representation of cystic fibrosis (*CF*) patients (<1% versus 25%). PMV was more frequent in PF patients (35.8%) than *CF* patients (22.1%) in the Hadem et al. study (4).

Our study identified higher BMI and diabetes mellitus in the recipient as independent risk factors for PMV occurrence. Surgical difficulties have been described in obese patients undergoing thoracic surgery (impaired visibility and accessibility to the operating site), which increased the duration of surgery (20). Obesity alters respiratory mechanics because of altered static compliance of the pulmonary and thoracic systems, increased resistance of the superior airways, and decreased functional residual capacity (21–23). Comorbidities linked to obesity (e.g., diabetes mellitus, high blood pressure, dyslipidaemia, renal disease, restrictive lung disease, sleep apnoea syndrome, and increased thrombotic risk) participate in the increased rate of postoperative complications (20). Overweight and obesity of the recipient are independent risk factors for grade 3 PGD occurrence in LT (24). Overweight was also associated with a lower 90-day survival rate of LT recipients (25). The BMI of the recipients in the Hadem study was not reported (4). Diabetes mellitus of the recipients is not an independent risk factor for PMV occurrence (*p* = 0.45).

Haemodynamic support by ECMO during surgery was significantly associated with PMV in multivariate analysis in our study. This finding is consistent with the Hadem et al. study, which described 72.6% of PMV in the recipients who required cardiopulmonary bypass or ECMO support during surgery versus 26.2% in the other patients (*p* < 0.001) (4).

Intraoperative transfusion of 5 RBC units or more was an independent risk factor for PMV in our study (p < 0.001). The number of intraoperative transfused RBC units was also higher in the PMV group in the Hadem study (p < 0.001) (4). Complications linked to transfusion and the susceptibility to prolonged MV (e.g., transfusion-related acute lung injury (TRALI) and transfusion-associated cardiac overload) have been largely described in the literature in various types of ICU patients (26, 27) and LT recipients (7, 28).

Mortality rates at day 90 and one-year were significantly higher in patients who received PMV (p < 0.001) in our study. These results are consistent with the Hadem et al. study, which described a one-year survival rate of 60% in PMV recipients versus 90% in non-PMV recipients (4). This result was comparable to the one-year survival in the PMV recipients of our cohort. Survival rates in patients who were discharged alive from the ICU and discharged alive from the hospital were identical regardless of PMV status in the Hadem et al. study. However, these mortality rates were not examined in our study.

Our study showed that PMV was significantly associated with all postoperative complications during hospitalisation in the ICU. These results are consistent with Hadem et al., who described an association between PMV and RRT requirement (*p* < 0.001), neurological complications after surgery (*p* = 0.001) or anastomotic dehiscence (*p* = 0.001) (4). These complications may be causes and consequences of PMV. Recipients who received PMV in our study suffered from significantly more infectious complications during their ICU stay (e.g., septic shock, number of pneumonia, and bacteraemia) than non-PMV recipients. PGD and grade 3 PGD were also significantly associated with PMV (48% of grade 3 PGD during the first 72 h in recipients with PMV versus 15% in the absence of PMV, *p* < 0,001). This association was observed in the Hadem study, which described a 25.3% rate of grade 3 PGD in patients with PMV versus 6.7% in the absence of PMV. The occurrence of PGD is likely a determining factor in the need for PMV.

Notably, tobacco use of the donor was not associated with PMV in our study. Tobacco use of the donor was not assessed in the Hadem study (4). However, published studies found a significant association between tobacco use of the donor and mortality at 30 and 90 days and 3 years (29). We hypothesise that the relatively small sample size of our cohort explains the lack of association between tobacco use of the donor and PMV occurrence.

Our study has several limitations. First, it was a monocentric study with a relatively small cohort. *CF* as an indication of LT was also very poorly represented in our cohort (2 (<1%) patients versus 10 to 20% according to the last ISHLT report (30)). Transplantation for *CF* is associated with a better outcome of the recipients (31), and it was associated with a lower rate of PMV in the Hadem et al. study (4). This particularity of our cohort may explain the relatively high incidence of PMV.

Notably, the characteristics of the third tertile of PMV recipients who received MV for >47 days were not different from the other PMV patients in our study (except for fresh frozen plasma and platelet transfusion during surgery). This result suggests that a higher duration of MV is not linked to a particular mechanism or specific subgroup of LT recipients.

## Conclusion

LT recipients who receive PMV after the surgical procedure are at high risk for one-year mortality and postoperative complications during the ICU stay. The identification of recipients with a high risk of PMV (high BMI and diabetes mellitus) before the surgical procedure may be helpful in the selection of recipients. Conditioning these recipients before surgery may have a positive impact on the postoperative course in the ICU.

## Data availability statement

The raw data supporting the conclusions of this article will be made available by the authors, without undue reservation.

## Ethics statement

The studies involving human participants were reviewed and approved by Paris North Hospital Institutional Review Board (Paris Diderot University, Assistance Publique Hôpitaux de Paris no. 0007477). Written informed consent for participation was not required for this study in accordance with the national legislation and the institutional requirements.

## Author contributions

EA participated in the study design, acquisition of data, analysis and interpretation of data, performed the statistical analyses, and drafted the manuscript. AB participated in the acquisition, analysis and interpretation of data. AD, SJ-B, ST, BL-J, LC, AR, JM, YC, and HM made substantial contributions to the interpretation of the data. CT and PM were involved in the study design, statistical analysis, interpretation of data, and drafting of the manuscript. All authors contributed to the article and approved the submitted version.

## Conflict of interest

JM received congress reimbursement fees from Biotest and CSLBehring.

The remaining authors declare that the research was conducted in the absence of any commercial or financial relationships that could be construed as a potential conflict of interest.

## Publisher’s note

All claims expressed in this article are solely those of the authors and do not necessarily represent those of their affiliated organizations, or those of the publisher, the editors and the reviewers. Any product that may be evaluated in this article, or claim that may be made by its manufacturer, is not guaranteed or endorsed by the publisher.

## Figures and Tables

**Table:** 

AKI	acute kidney injury
BAL	bronchoalveolar lavage
BMI	body mass index
COPD	chronic obstructive pulmonary disease
ECMO	extracorporeal membrane oxygenation
HBP	high blood pressure
ICU	intensive care unit
PF	pulmonary fibrosis
LT	lung transplantation
MV	mechanical ventilation
NBA	neuromuscular blocking agent
NIV	non-invasive ventilation
PGD	primary graft dysfunction
PMV	prolonged mechanical ventilation
PRC	packed red cell
RRT	renal replacement therapy
SAPS II	Simplified acute physiology score II
SOFA	sequential organ failure assessment
