# Supplementary material for: Prolonged mechanical ventilation after lung transplantation: risks factors and consequences on recipient outcome
Source: Front Med (Lausanne). 2023 May 9;10:1160621. doi: 10.3389/fmed.2023.1160621 (PMC10203407; doi:10.3389/fmed.2023.1160621)
Supplement: Supplementary file 1 [file Table_1.DOCX]

**SUPPLEMENTAL DATA**

**TABLE S1:** Risk factors for MV> 47 days, univariate analysis

|  | | | **MV 15-47 days,**  **n = 43 (67%)** | **MV >47 days,**  **n = 21 (33%)** | **p** | | |
| --- | --- | --- | --- | --- | --- | --- | --- |
| **Preoperative variables** | |  |  |  |  | |  |
| Age (recipient), years, median [IQR] | | 56 [46-59] | 55 [45-60] | 0.94 | | |  |
| Male gender, n (%) | | 24 (56) | 14 (67) | 0.41 | | |  |
| Diagnosis leading to LT, n (%) | |  |  |  | | |  |
| - Pulmonary fibrosis | | 13 (30) | 10 (48) | 0.17 | | |  |
| - COPD | | 10 (23) | 2 (9.5) | 0.31 | | |  |
| BMI, median [IQR] | | 25 [21-28] | 27 [24-30] | 0.14 | | |  |
| BMI > 30 kg/m2 | | 7 (16) | 6 (29) | 0.32 | | |  |
| Comorbidities | |  |  |  | | |  |
| - High blood pressure, n (%) | | 9 (21) | 8 (38) | 0.14 | | |  |
| - Pulmonary hypertension, n (%) | | 17 (42) | 10 (48) | 0.70 | | |  |
| - Diabetes mellitus, n (%) | | 5 (12) | 6 (29) | 0.16 | | |  |
| - Dyslipidaemia, n (%) | | 7 (16) | 2 (9.5) | 0.71 | | |  |
| Serum albumin, median [IQR] | | 39 [37-41] | 37 [33-44] | 0.52 | | |  |
| Serum creatinine, median [IQR] | | 65 [52-81] | 72 [63-87] | 0.29 | | |  |
| Re-transplantation, n (%) | | 1 (2) | 2 (10) | 0.26 | | |  |
| High emergency LT, n (%) | | 10 (23) | 8 (38) | 0.22 | | |  |
| ECMO support before surgery, n (%) | | 4 (9) | 5 (24) | 0.14 | | |  |
| MV before surgery, n (%) | | 4 (9) | 1 (5) | >0.999 | | |  |
| High flow oxygen therapy before surgery, n (%) | | 9 (21) | 8 (38) | 0.14 | | |  |
| **Donors variables** | |  |  |  | | |  |
| Male gender, n (%) | | 20 (47) | 13 (62) | 0.61 | | |  |
| PaO2/FiO2 ratio, median [IQR] | | 390 [328-444] | 376 [330-408] | 0.47 | | |  |
| Age (donor), median [IQR] | | 55 [42-61] | 55 [50-60] | 0.66 | | |  |
| Tobacco use (donor), n (%) | | 21 (50) | 9 (47) | 0.85 | | |  |
| Transfusion of the donor, n (%) | | 13 (31) | 6 (30) | 0.94 | | |  |
| Duration of MV, median [IQR] | | 2 [2-3.5] | 2 [1-6] | 0.99 | | |  |
| **Intraoperative variables** | |  |  |  | | |  |
| Bilateral LT, n (%) | | 35 (81) | 14 (67) | 0.22 | | |  |
| Duration of anaesthesia, min, median [IQR] | | 420 [400-520] | 380 [360-458] | 0.19 | | |  |
| Epidural anaesthesia, n (%) | | 24 (56) | 13 (65) | 0.49 | | |  |
| Haemodynamic support by ECMO, n (%) | | 38 (88) | 19 (90) | >0.999 | | |  |
| Norepinephrine or epinephrine > 0.5 µg/kg/min, n (%) | | 23 (55) | 9 (43) | 0.37 | | |  |
| Red blood cell transfusion, n (%) | | 37 (86) | 14 (67) | 0.099 | | |  |
| Transfusion > 5 PRC, n (%) | | 16 (37) | 6 (29) | 0.50 | | |  |
| Fresh frozen plasma transfusion, n (%) | | 37 (86) | 10 (48) | 0.001 | | |  |
| Platelet transfusion, n (%) | | 17 (40) | 3 (14) | 0.041 | | |  |
| **At admission in ICU** | |  |  |  | |  |  |
| SOFA | 8 [7-10] | 7 [5-8] | 0.062 | | |  |  |
| SAPS II | 43 [32-51] | 44 [36-57] | 0.74 | | |  |  |
| Lactate > 3 mmol/L | 22 (51) | 6 (29) | 0.087 | | |  |  |
| Lactate > 2 mmol/L | 30 (70) | 12 (57) | 0.32 | | |  |  |

| BMI: Body mass index; COPD: chronic obstructive pulmonary disease; ECMO: Extra-Corporeal Membrane Oxygenation; HBP: High blood pressure; ICU: intensive care unit; LT: lung transplantation; MV: mechanical ventilation; OR: odds ratio; PMV: prolonged mechanical ventilation; PRC: Packed red cell; SOFA: sequential organ failure assessment; SAPS: simplified acute physiology score. |
| --- |

**TABLE S2:** Postoperative complications and outcome of the third tertile of PMV recipients, univariate analysis

|  | **MV 15-47 days**,  N = 43 (67%) | **MV > 47 days**,  N = 21 (33%) | | p |  |
| --- | --- | --- | --- | --- | --- |
| **Postoperative complications** |  |  | |  |  |
| Multiorgan failure syndrome, n (%) | 24 (56) | 17 (81) | | 0.049 |  |
| Median duration of catecholamine support, days, median [IQR] | 4 [2-12] | 6 [3-12] | | 0.34 |  |
| Median duration of ECMO support, days, median [IQR] | 1 [0-3] | 1 [0-4.5] | | 0.39 |  |
| Septic shock, n (%) | 28 (65) | 17 (81) | | 0.19 |  |
| Number of pneumonia, , median [IQR] | 2 [1-3] | 3 [3-4] | | 0.004 |  |
| Bacteriemia, n (%) | 13 (31) | 6 (30) | | 0.94 |  |
| Prone positioning, n (%) | 16 (37) | 12 (57) | | 0.13 |  |
| PGD during the first 72 hours, n (%) |  |  | | 0.012 |  |
| Grade 1 | 3 (7.0) | 0 (0) | |  |  |
| Grade 2 | 2 (4.7) | 6 (29) | |  |  |
| Grade 3 | 27 (63) | 7 (33) | |  |  |
| Grade 3 PGD at H24, n (%) | 19 (44) | 6 (29) | | 0.23 |  |
| Grade 3 PGD at H48, n (%) | 16 (37) | 6 (29) | | 0.50 |  |
| Grade 3 PGD at H72, n (%) | 14 (33) | 6 (29) | | 0.75 |  |
| Neuromuscular blocking agent administration, n (%) | 28 (65) | 18 (86) | | 0.085 |  |
| Atrial fibrillation, n (%) | 20 (47) | 14 (67) | | 0.13 |  |
| AKI, n (%) | 30 (70) | 16 (76) | | 0.59 |  |
| Renal replacement therapy, n (%) | 10 (23) | 3 (14) | | 0.52 |  |
| Thoracic surgical reintervention, n (%) | 18 (42) | 9 (43) | | 0.94 |  |
| Abdominal surgery, n (%) | 11 (26) | 3 (14) | | 0.36 |  |
| Antibody mediated rejection, n (%) | 20 (47) | 11 (52) | | 0.66 |  |
| Acute cellular rejection, n (%) | 11 (26) | 5 (24) | | 0.88 |  |
| **Outcome** |  |  | |  |  |
| Duration of MV, median [IQR] | 27 [21-34] | 72 [53-77] | | <0.001 |  |
| Duration of ICU stay, median [IQR] | 40 [32-53] | 79 [74-103] | | <0.001 |  |
| Death at day 90, n (%) | 9 (21) | 5 (24) | >0.999 | | |
| Death at one year, n (%) | 16 (37) | 12 (57) | 0.13 | | |

| AKI: acute kidney injury; ECMO: extracorporeal membrane oxygenation; ICU: intensive care unit; PMV: prolonged mechanical ventilation; PGD: Primary graft dysfunction; RRT: renal replacement therapy. |
| --- |
